# Development and validation of a patient reported experience measure for experimental cancer medicines (PREM-ECM) and their carers (PREM-ECM-Carer)

**DOI:** 10.1186/s12885-024-11963-x

**Published:** 2024-04-19

**Authors:** Chelsea S. Sawyer, Sally Taylor, Louise Carter, Melissa Stanworth, Michelle Davies, Fiona Thistlethwaite, Jo Taylor, Charlotte Eastwood, Janelle Yorke

**Affiliations:** 1 Division of Psychology and Mental Health, The University of Manchester, Manchester, UK; 2 Christie Patient Centred Research (CPCR), The Christie NHS Foundation Trust, Manchester, UK; 3 NIHR Manchester Clinical Research Facility, The Christie NHS Foundation Trust, Manchester, UK; 4 Division of Cancer Sciences, School of Medical Sciences, Faculty of Biology Medicine & Health, University of Manchester, Manchester, UK; 5 The Experimental Cancer Medicine Team, The Christie NHS Foundation Trust, Manchester, UK; 6 Division of Nursing Midwifery and Social Work, School of Health Sciences, Faculty of Biology Medicine & Health, University of Manchester, Manchester, UK; 7 The Christie NHS Foundation Trust, Manchester, UK; 8 School of Nursing, The Hong Kong Polytechnic University, Hong Kong, China

**Keywords:** Clinical trials, Patient experience, Questionnaire development, Carers, Rasch analysis, Cancer

## Abstract

**Background:**

Our aim was to develop a validated Patient Reported Experience Measure (PREM) to capture patient and carer experience during participation in experimental cancer medicine trials (ECM): called PREM-ECM.

**Methods:**

Mixed method design, consisting of four stages. Questionnaire items were produced for both patients and carers using interviews, focus groups, and cognitive interviews with patients and carers separately. For both patient and carer PREMs, a cross-sectional questionnaire study was conducted to identify final items for inclusion using hierarchical item reduction and Rasch analysis. Questionnaire validity and reliability were assessed, including administration feasibility.

**Results:**

Initial interview participants suggested the need for three PREMs, two specific to patients: (i) a ‘prior’ questionnaire that captured experiences of trial introduction, screening, consenting, and early trial experience (< 6 weeks post consent); and (ii) ‘on-trial’ that captured experiences of ongoing consent and trial participation; and (iii) a PREM specific for carers. The draft 25-item ‘prior’ questionnaire was completed by 162 patients and 162 patients completed the draft 35-item ‘on-trial’ questionnaire. Hierarchical and Rasch analysis produced a 14-item ‘prior’ list and a 15-item list for ‘on-trial’. Both patient PREM’s demonstrated a good fit to the Rasch model following Bonferroni correction (X^2^p = 0.008). The carer 34-draft item questionnaire was completed by 102 participants. Hierarchical and Rasch analysis produced a 13-item list for PREM-ECM-Carer, with good fit to the Rasch model ( X^2^*p* = 0.62). The pilot testing demonstrated the feasibility of all the PREMs in capturing patient and caregiver experiences in routine clinical settings.

**Conclusions:**

The three PREM-ECM questionnaires will be the first validated experience measures for ECM trial patients and their carers. These questionnaires may be used to assess patients’ and their carers’ experiences of ECM and enable robust comparisons across cancer trial units highlighting areas for service improvement.

**Supplementary Information:**

The online version contains supplementary material available at 10.1186/s12885-024-11963-x.

## Background

Clinical trials are a critical element of cancer research and care. The aim of clinical trials are to test new treatments before they are adopted routinely into clinical practice [1]. Clinical trials include early phase clinical trials (phases 1 and 2) which test the safety, pharmacokinetics and pharmacodynamics of novel drugs, and later phase trials (phases 3 and 4) that determine the efficacy of novel treatment [2, 3]. Early phase cancer medicine trials (ECM) have been found to be emotionally and practically burdensome for trial participants, and can lead to reduced quality of life [4–6]. In the initial phases of clinical trials (Phases 1 and 2), the primary focus centres on evaluating safety and determining the appropriate dosage therefore participants may encounter unknown risks and potential side effects. In Phase 3 trials the most effective dosage for administering the drug is typically well-established and the trials often focus on comparing the new treatment with standard treatments. Given the different aims and focus of early phase and phase 3 trials, the experiences of participants are likely to be very different. In addition, although the ECM experience can present challenges for patients and participants often report that they view the trial as ‘their last chance”, participation also offers hope, feelings of altruism and increasingly therapeutic benefit [6, 7]. Involvement in ECM can affect not only the patient’s but also their family’s quality of life, potentially necessitating adjustments in daily routines and social activities as family and friends take on caregiving roles [8–12].

The concept of patient experience incorporates respect for person-centred values, preferences and needs, integration of care, social and emotional support, involvement of family and friends, information giving, shared-decision making and practical comfort [13]. Assessing patient experience is central to understanding and improving the care patients receive. Patient experiences can be collected in various ways; however, the most frequently adopted method is satisfaction surveys. More recently questionnaires termed Patient Reported Experience Measures (PREMs) have been developed and validated using appropriate psychometric approaches. PREMs measure the patient’s experience of the care received, which includes the physical and emotional support, transition and continuity, and respect for patient-centered values, and are deemed to provide more detailed information compared with satisfaction surveys [14, 15]. The routine clinical use of PREMs can identify areas for improvement and have the potential to improve the patient’s quality of life [16]. Furthermore, PREMs to date focus on the person receiving health care, the patient, with little focus on the experience of their informal caregiver. This is important as carers are often pivotal to providing support to cancer patients [17] and in the context of cancer clinical trials the carer role can viewed as burdensome and distressing [18].

Validated PREMs are increasingly being used to assess patient experience across different patient groups and healthcare facilities. PREMs can be generic such as the Patient Experience Questionnaire (PEQ) [19] which was developed in primary care and explores doctor patient communication. PREMs have also been developed for patients with health conditions such as cancer, Chronic Obstructive Pulmonary Disease (COPD), and musculoskeletal disorder [20–23]. Measuring the healthcare experience of specific diagnostic groups enables audit and benchmarking across similar services. At present, very little patient experience data is available to capture the experiences of patients and their carers taking part in clinical trials. The data collected would be beneficial to improve patient and carer experiences and the services available to them. Our aim was to develop and validate a PREM to measure patient and carer experience in participating in an ECM trial.

## Methods

### Study design

This study used a mixed-method design, consisting of four stages. This methodology was considered appropriate as it is an established research methodology for PREM development which has been utilised in previous similar studies [23–25]. The Good Reporting of A Mixed Methods Study (GRAMMS, see appendix 1) was used to report the study methodology. Stage I (item generation) and Stage II (cognitive debriefing) were qualitative and informed the draft item list and questionnaire layout. Stage III (item reduction and psychometric analyses) and Stage IV (pilot testing) were cross-sectional quantitative designs, used to refine the item lists.. The inclusion criteria for patients were (a) patients over the age of 18 years who were screened for an ECM trial (phases 1 or 2) and (b) a diagnosis of any cancer type. Inclusion criteria for carers were family/friends of patients (a) who have been screened for participation in an ECM trial, and (b) any cancer type. Patients and carers were excluded if they were unable to provide informed consent, or comprehend written English. For each study stage participants were recruited from a regional cancer centre Northwest England. The clinical trial research team approached potential participants, who were provided with written study information and contact details for the PREM development team. Ethical approval was gained from the insert Research Ethics Committee (18/SC/0299) and the local NHS Trusts. In all four stages patients’ demographic and clinical information were obtained from their medical record, after the interview or questionnaire was completed, with the patients informed consent. Demographic information was obtained from carers directly.

#### Stage I: item generation

We aimed to recruit approximately 30 patient participants for Stage 1. Participants were interviewed face-to-face either at their home or in a quiet hospital room, depending on patient preference. Both the interviews and focus groups used the same topic guide (see appendix 2) and were audio recorded, with the participant’s written informed consent. The patient topic guide was used to capture (i) decision-making support regarding participating/declining the trial, (ii) experiences of their current and/or previous participation in clinical trials, (iii) the provision of trial information, delivery of the trial and; (iv) their expectation and experience of the trial treatment, such as side-effects.

From the interviews and initial focus groups key themes and potential items for the questionnaire were extracted. These themes were discussed in a second focus group with participants who suggested the need for two questionnaires, (i) one that focused on the early screening and consenting processes and (ii) focusing on the experience during on-going trial participation. Therefore, two patient draft PREM-ECMs were created: PREM-ECM-prior and PREM-ECM-on-trial. We also then developed a carer topic guide (see appendix 3) to capture (i) carers involvement with patients decision to participate on trials, (ii) their experiences of the patient participating in the trial and caring for patient, (iii) impact of patients participation in the trial on them. We aimed to recruit approximately 15 carer participants. From the themes extracted from the interviews one carer draft PREM-ECM was created.

#### Stage II: cognitive debriefing

We aimed to recruit 10 patients and 5 carers for Stage II. Face to face cognitive debriefing interviews were conducted in a quiet hospital room. Cognitive interviewing was employed as a methodological approach to assess the suitability of questionnaire items for their intended purpose. This process was carried out prior to the administration of the questionnaire to patients and carers, aiming to ensure clarity and comprehension of the items [26]. Participants were presented with a preliminary set of questions and encouraged to engage in a ‘think-aloud’ exercise, articulating their thoughts while responding to the questionnaire. After this activity the interviewer addressed any items that were misunderstood, and participants were prompted to rephrase questions for improved clarity. Additionally, participants reviewed several Likert scales with different response options, during which they vocalized their thought processes while answering questions associated with each scale.

#### Stage III: item reduction

***PREM-ECM-prior.*** Patients who were approached about participating in a clinical trial but were not suitable for a clinical trial (also referred to as a ‘screen failure’), declined participation in a trial or had been participating in a trial for less than six weeks were asked to complete the PREM-ECM-prior.

***PREM-ECM-on-trial.*** Patients who have been on the trial for more than six weeks were asked to complete the PREM-ECM-on-trial. If a patient had previously completed the PREM-ECM-prior then they were also invited to complete the PREM-ECM-on-trial section. In stage III, the method detailed below was used for both questionnaires.

In addition to the relevant PREM-ECM, participants completed additional questionnaires to enable construct validity testing (these assess whether the questionnaire captures what it is meant to measure [27]) these included:

Patient satisfaction with cancer care questionnaire [EORTC PATSAT-C33 [28]]: The EORTC PATSAT-C33 is a 33 item questionnaire. The questionnaire includes three sections addressing doctors, nurses/radiotherapy technicians, and service and care organisation. Participants are asked to rate their experience for each item from 1 to 5 (poor to excellent).

Patient Experience Questionnaire (PEQ; [19]): The PEQ is an 18 item questionnaire exploring five key areas: outcome of the visit; communication experiences, communication barriers; experience with health care staff and emotions after visit.

Hospital Anxiety and Depression Scale (HADS [29]): the HADS is a 14-item scale that assesses psychological distress with two sub scales: 1) anxiety (0–21) and depression (0–21), higher scoring denoting worse psychological distress. The HADS was used to explore associations with PREM-ECM.

A sub-sample of participants were invited to complete the draft PREM-ECM-prior/on-trial approximately one week later, to determine test-retest reliability (this measures the stability of the questionnaire over time, to ensure participants score similarly at two separate time points [30]).

### Carers

Family or friends of patients recruited onto a trial were requested to complete the draft PREM-ECM-Carer. Additionally, carers were asked to fill out two other validated questionnaires: one assessing caregiver-reported experience and the other assessing psychological well-being (HADS). The Adult Carer Quality of Life Questionnaire (AC-QoL) is an 80-item survey that evaluates the quality of life for caregivers across eight domains: support for caring, caring choice, caring stress, financial matters, personal growth, sense of value, ability to care, and caregiver satisfaction. A subset of carers completed the PREM-ECM-Carer draft approximately one week later to assess test-retest reliability.

#### Stage IV: pilot testing

To assess the feasibility of using the PREMs in research clinics, patients and their family and friends who did not take part in any of the early development stages were invited to complete the questionnaire during a routine trial visit. We aimed to recruit approximately 10 patients and 10 carers to complete each questionnaire.

### Data Analysis

In stage I an inductive thematic approach was used [31]. Transcripts were analysed to determine the patterns and themes across the interviews and focus groups, which themes were frequently reported and the importance of topics to individuals. Once themes had been determined, the most articulate quote capturing the theme/aspect of the patients experience, was selected as the potential item.

In stage II the feedback and annotations on the questionnaires were coded, each question was coded as either no changes needed, needs improving/refining, or item not applicable/relevant.

In stage III hierarchical methods were applied to identify items for potential removal. This included flagging items if they demonstrated a floor/ceiling effect (set at ≥ 80%). Mann Whitney U-tests were performed to determine if there was any gender bias and Spearman correlation tests determined if there was any age bias. Spearman correlation tests were also performed to determine correlations between individual items and item-total score for each of the PREM-ECM questionnaires.

The remaining items were further analysed using Rasch analysis to identify items with good measurement properties and to assess the overall fit to the Rasch unidimensional model indicated by a non-significant chi-square statistic ( *p* > 0.05) [32]. To reduce the risk of Type 1 error, due to the high number of comparisons, Bonferroni corrections were made; overall fit to the Rasch model for both questionnaires was set at p >0.003. A Principals Components Analysis (PCA) using varimax rotation was used to assess the underlying structure of the final item set. The number of components extracted was based on eigenvalues and allocation of an item to a component was determined by a factor loading which by convention is set at > 0.5 [33].

The Cronbach’s alpha coefficient was used to test the internal reliability of each questionnaire and any sub-scales, where α ≥ 0.70 demonstrating acceptable internal consistency. Intra-Class Correlation Coefficient (ICCC) was used to assess reliability over time (7 day period) [34]. All statistical analyses were conducted using IBM Statistical Package for Social Sciences (SPSS) Version 25 or RUMM2030, with a *p* < 0.05 for statistical significance.

In stage IV descriptive analysis was used to describe participants demographic and clinical background.

## Results

**Fig. 1:**
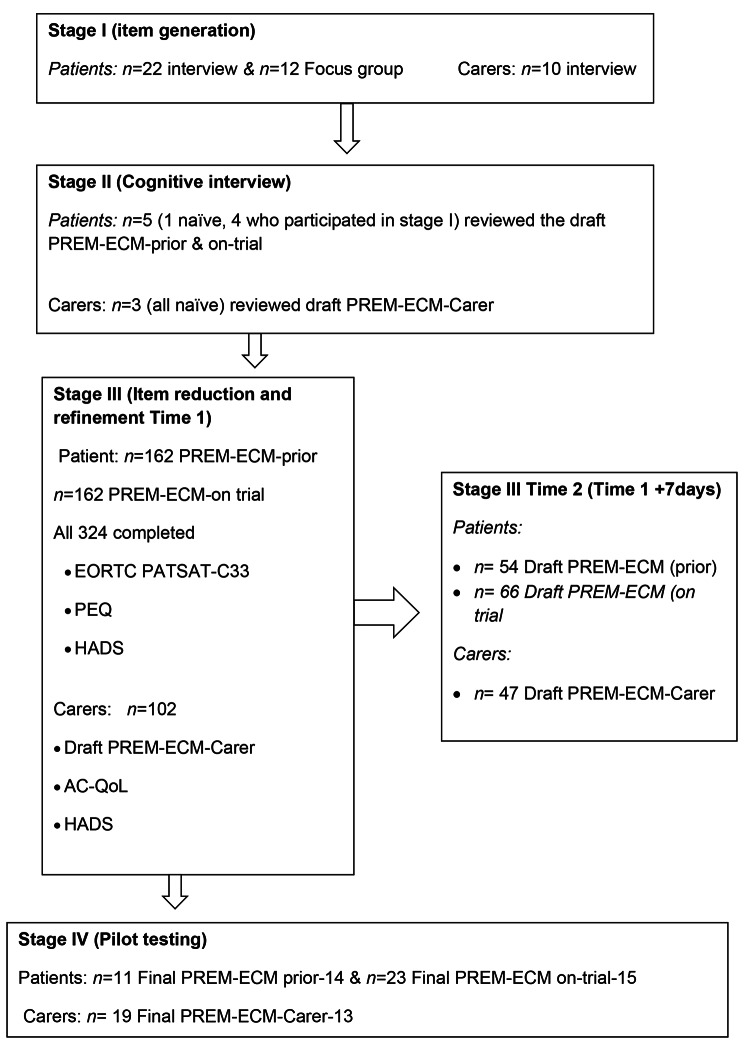
Study schema

Figure 1 presents the number of patients and carers who were approached and recruited onto each of the study stages. In total, 34 patient participants were recruited in stage I and five patient participants, one who was naive to the study (i.e. had not participated in any of the study’s earlier stages), were recruited in stage II. In total 324 patient participants were recruited in stage III; 162 completed the ‘prior-trial’ draft questionnaire and 162 completed the ‘during-trial’ questionnaire. Thirty participants completed both stage III questionnaires. Additional patient participants were recruited in stage IV; 25 completed the PREM-ECM-prior-14 and the PREM-ECM-on-trial-15 questionnaires. In total, ten carer participants were recruited in stage I and three study naïve carer participants were recruited in stage II. In total 102 carer participants were recruited in stage III and 19 were recruited into Stage IV. Participants’ demographics and clinical demographics for each of the stages are described in Tables 1 and 2. Carer participants’ demographics are described in Supplementary Table 1.

**Table 1:** Demographic characteristics of patient participants for each stage of the study

	Stage I		Stage II	Stage III		Stage IV	
	Interviews (*n* = 22)	Focus groups (*n* = 12)	*Cognitive interview (n* = 5)	PREM-ECM Prior *(n* = 162)	PREM-ECM on trial (*n* = 162)	PREM-ECM prior-14 *(n* = 11)	PREM-ECM on-trial-15 *(n* = 23)
**Age Mean (SD)**	63.64 (10.16)	67.92 (7.55)	66.00 (5.51)	59.11 (11.72)	61.48 (11.40)	62.23 (10.62)	65.26 (7.78)
**Age range (years )**	42–83	52–79	59–73	30–88	27–87	41–77	47–80
**Gender (Male)** %(*n*)	45.45% (10)	91.67% (11)	100.00% (5)	47.5% (77)	52.5% (85)	54.5% (6)	47.8% (11)
**Ethnicity**							
White British %(*n*)	95.45% (12)	100.00% (12)	100.00% (5)	91.25% (146)	91.4% (148)	100% (11)	100% (23)
Irish %(*n*)	-	-	-	3.13% (5)	1.85% (3)	-	-
White other	-	-	-	1.88% (3)	-	-	-
Indian	-	-	-	0.63% (1)	1.23%(2)	-	-
Chinese	4.55% (1)	-	-	1.88% (3)	0.62% (1)	-	-
Any other Asian background	-	-	-	-	1.23% (2)	-	-
African	-	-	-	0.63% (1)	1.85% (3)	-	-
Caribbean	-	-	-	-	1.23% (2)	-	-
White & Black African	-	-	-	-	0.62%(1)	-	-
White& Asian	-	-	-	0.63% (1)	-	-	-
**Marital status**							
Single	4.55% (1)	16.67% (2)	20.00% (1)	8.0% (13)	10.5% (17)	-	19.0% (4)
Married/domestic partner	90.90% (20)	66.67% (8)	60.00% (3)	80.9% (131)	80.9% (131)	100% (9)	66.7% (14)
Widowed	4.55% (1)	8.33% (1)	20.00% (1)	4.3% (7)	4.3% (7)	-	-
Divorced	-	8.33% (1)	-	4.9% (8)	3.7% (6)	-	14.3% (3)
Separated	-	-	-	0.9% (2)	0.6% (1)	-	-
**Employment status**							
Employed	-	-	-	25.2% (39)	18.2% (29)	44.4% (4)	23.8% (5)
Self-employed	-	8.33% (1)	25.00% (1)	1.9% (3)	6.3% (10)	-	4.8% (1)
Student	-	-	-	-	0.6% (1)	-	-
Retired	72.72% (16)	83.33% (10)	75.00% (3)	52.3% (81)	51.6% (82)	55.6% (5)	57.1% (12)
Unable to work	18.18% (4)	8.33% (1)	-	18.1% (28)	22.0% (35)	-	9.5% (2)
Home maker	9.1% (1)	-	-	2.6% (4)	1.3% (2)	-	4.8% (1)

**Table 2:** Clinical demographic of patient participants for each stage of the study

	Stage I				Stage II		Stage III		Stage IV		
	Interviews (*n* = 22)	Focus groups (*n* = 12)			*Cognitive interview (n* = 5)	PREM-ECM prior *(n* = 162)		PREM-ECM on-trial (*n* = 162)		PREM-ECM (prior-14 *(n* = 11)	PREM-ECM on-trial-15 (*n* = 23)
**Performance status**											
0%(*n*)	52.94% (9)		45.45% (5)	16.67% (1)		48.48% (64)		48.77% (79)		54.54% (6)	59.1% (13)
1%(*n*)	47.06% (8)		54.55% (6)	66.67% (4)		51.52% (68)		51.23% (83)		45.45% (5)	40.9% (9)
**Participated in a trial (phase 1–3) before % (n)**	22.73% (5)		25.00% (3)	20.00% (1)		15.43% (25)		20.99% (34)		0%	17.39% (4)
**Trial Phase**											
Screen fail	9.09% (2)	-			-	5.56% (9)		-		-	-
Decline to participate in trial	-	-			-	0.62% (1)		-		-	-
Phase 1	50.00% (13)	50% (6)			40% (2)	45.06% (72)		41.36% (67)		45.5% (5)	34.78% (8)
Phase 2	31.82% (7)	50% (6)			60% (3)	44.44% (72)		58.64% (95)		54.5.% (6)	65.22% (15)
Non-treatment trial (i.e. Target molecular)	9.09% (2)	-			-	4.32% (7)		-		-	-
**Time on trial (**< 1 year)	54.55% (12)	50% (6)			40% (2)	100% (162)		76.54% (124)		100% (11)	8.69% (2)
**Disease group**											
Breast	31.82% (7)	-			-	17.90% (29)		21.6% (35)		9.09% (1)	-
Brain	-	-			-	17.90% (29)		6.80% (11)		-	-
Lower GI	13.64% (3)	16.67% (2)			-	12.97% (21)		6.20% (10)		9.09% (1)	8.70% (2)
Upper GI	-	-			-	5.65% (9)		2.47% (4)		27.27% (3)	17.39% (4)
Gynaecology	-	-			-	9.26% (16)		-		18.18% (2)	30.43% (7)
Head & Neck	-	8.33% (1)			-	6.79% (11)		3.70% (6)		9.09% (1)	
Haematological	9.09% (2)	8.33% (1)				1.23% (2)		4.94% (8)		9.09% (1)	13.04% (3)
HPB					20.00% (1)	3.70% (6)		1.24% (2)		-	-
Lung	22.73% (5)	16.67% (2)			20.00% (1)	19.14% (31)		16.67% (27)		9.09% (1)	
Lymphoma	18.18% (4)	41.67% (5)			60.00% (3)	4.94% (8)		9.25% (15)		-	8.70% (2)
Melanoma	-	-			-	1.85% (3)		2.47% (4)		-	4.35% (1)
Genitourinary (GU, excluding prostate)	4.55% (1)	8.33% (1)			-	-		1.85% (3)		-	-
Prostate	-	-			-	7.41% (12)		4.94% (8)		-	-
Sarcoma	-	-			-	1.23% (2)		0.62% (1)		9.09% (1)	17.39% (4)
Thymus	-	-			-	-		0.62% (1)		-	-
Unknown primary source	-	-			-	3.09% (5)		2.47% (4)		-	-

### Stage I: item generation

#### Patients

Twenty-two patients were interviewed. The interviews ranged from 14 to 62 minutes. To ensure we captured the experiences of patients in early-phase clinical trials, we deliberately recruited individuals with diverse diagnoses, recognising the variability in treatments. Our efforts extended to encompass a variety of early-phase trial types, ensuring a comprehensive representation of their collective experiences. We aimed for an equitable distribution across gender and performance status, which influence patient’s ability to function daily and care for themselves. As well as a diverse, range of ages. After the last three interviews yielded no new themes, signalling data saturation, we proceeded to invite twelve study naive patients to attend one of the two face-to-face focus groups. The focus groups ranged from 48 to 108 minutes.

Interviews were conducted first to gain a deep understanding of individual’s experiences on a clinical trial. The main themes identified were shared decision making, provision of information, confidence to decline or withdraw from the trial, trial burden, side effects, personalised care, options for treatment, and emotional support for self/family/friends (supplementary Table 2 presents the themes and their sub-themes with supporting quotations). Once these themes were generated, we held two focus groups. In this setting, participants engaged in open discussions and reflections on the identified themes, offering a unique opportunity for them to share, compare, and contrast their individual experiences within the context of the broader thematic framework. During the second patient focus group, the need for two separate PREM-ECMs was highlighted due to the different stages in a clinical trial. Participants suggested that one questionnaire should capture patients’ initial introduction to the trial, the screening process, and decision-making regarding consenting to the trial. Participants stated that the second questionnaire should focus on the impact of participating in the trial on the patient and their family/friends and decision-making regarding continuing/ withdrawing from the trial again. Participants agreed that the second questionnaire should be administered after at least one cycle of treatment or at least six weeks, to enable patients to have experienced the trial process, side-effect and trial follow-up.

The combination of individual interviews and focus groups not only allowed for a comprehensive exploration of personal narratives but also facilitated a collective sense-making process, enriching the depth and validity of our study findings.

#### Carers

Ten carers were interviewed, interviews ranged from 32 to 63 minutes. After the last three interviews yielded no new themes, we stopped data collection. The main themes identified were coping with uncertainty around the trial, requiring more information about patients progress, updates on trial progress and support available (financial, psychological and practical), confidence disclosing information, shared decision making, trial burden, managing side-effects, and practical, emotional and financial support for self/family/friends (supplementary Table 3 presents the themes and their sub-themes with supporting quotations).

### Stage II: cognitive debriefing

#### Patients

Five patients participated in a face-to-face cognitive interview. Four of the patients had participated in stage I and one participant was study naive. Initially, three out of the five preferred the traditional survey layout (responses on a scale of ‘strongly disagree’ to ‘strongly agree’) whereas two preferred the semantic layout (with opposing statements presented either side of a numbered 1–5 scale). However, one person reflected that the traditional layout did not ‘make them really think about the question’ whereas the semantic layout made them stop and think about each question and how they might answer. As such, the semantic layout was used.

All five participants reviewed the 27 and 39 draft items for the PREM-ECM ‘prior’ and ‘on-trial’, respectively. Participant feedback resulted in six items being modified and two items removed from the ‘prior’ item list, producing 25 draft items. For the ‘on-trial’ PREM-ECM, eight items were modified and four items removed, producing 35 draft items. The draft PREMs were then reviewed by our study patient representative and the research team with small changes to the wording and item order being made.

#### Carers

All three carers were study naïve and reviewed the 37 draft items. Following their feedback, nine items were modified and three were removed, producing 34 draft items. The draft list underwent a review process involving both the patient representative and the research team, resulting in minor adjustments to the wording and item sequence.

### Stage III: item reduction

#### Patients

##### PREM-ECM-prior

One hundred and sixty-two participants completed the PREM-ECM-Prior. Twenty-five items were included in the draft list and six of these were removed in hierarchical reduction: ceiling effect (*n* = 6), gender bias (*n* = 1), and item-to-item correlation (*n* = 6); some items were removed for multiple reasons (see Table 3). Rasch analysis was conducted with the remaining 19 items. Five items were removed due to poor fit to the Rasch unidimensional model (see Table 3). The final 14-item solution, after Bonferroni correction, demonstrated good fit to the Rasch model (*p* = 0.008; PSI = 0.75) and good distribution of item scores (logit range: +0.05 to + 0.25).

**Table 3:** Reason for item removal for PREM-ECM-prior

Question Number	Question (High Score Answer)	Ceiling > 79%	gender bias	Age Correlation	Correlation with other items	Rasch
7	I am given enough time to ask questions	X			X	
8	I understood what I was consenting to	X			X	
9	I feel comfortable asking the research team any questions	X			X	
16	Enough time to decide participate	X	X		X	
18	I am involved as much as I want to be with the decision to take part in the trial	X			X	
21	The research team treats me as an individual	X			X	
2	I understand the information about the trial					X
6	There is enough time to read and think about the trial information					X
14	I do not feel the research team has built up my hopes for positive results					X
15	My concerns about taking part in the trial have been fully addressed					X
17	There is enough time to decide whether I want to take part in the trial					X

Each PREM-ECM-prior item is scored on a differential scale with opposing adjectives from a score of 0 (describing a poor experience) to 5 (describing a good experience); the total scores range from 0 to 70. The mean and range of scores for each of the final 14 items are presented in Table 4, with Total PREM-ECM-Prior scores ranging from 1 to 65.

**Table 4:** The mean and range of scores for each item of the PREM-ECM prior-14 & PREM-ECM on-trial-15 & PREM-ECM-Carer-13

Prior-14 Items [Original number]	Mean item score (SD)	Score range	on-trial-15 Item [Original number]	Mean Item score (SD)	Score range	Carer-13 Items [Original number]	Mean item score (SD)	Score range
1 [1]	4.53 (0.75)	1–5	1 [5]	4.69 (0.67)	1–5	1 [9]	4.38 (1.05)	0–5
2 [3]	3.93 (1.48)	0–5	2 [4]	4.53 (1.09)	0–5	2 [12]	4.50 (1.03)	0–5
3 [4]	4.45 (1.18)	0–5	3 [10]	4.82 (0.59)	0–5	3 [13]	4.54 (1.09)	0–5
4 [5]	4.53 (1.08)	0–5	4 [14]	4.40 (1.07)	0–5	4 [14]	4.30 (1.42)	0–5
5 [10]	4.18 (1.46)	0–5	5 [21]	4.83 (0.60)	0–5	5 [16]	4.32 (1.26)	0–5
6 [11]	4.61 (0.95)	0–5	6 [22]	4.30 (1.16)	0–5	6 [18]	4.38 (1.26)	0–5
7 [12]	4.42 (1.16)	0–5	7 [27]	4.43 (1.08)	0–5	7 [19]	3.99 (1.35)	0–5
8 [13]	4.36 (1.14)	0–5	8 [28]	4.65 (0.79)	1–5	8 [22]	3.87 (1.48)	0–5
9 [19]	4.54 (1.12)	0–5	9 [33]	3.99 (1.32)	0–5	9 [23]	3.81 (1.52)	0–5
10 [20]	4.65 (0.93)	0–5	10 [34]	4.18 (1.16)	0–5	10 [26]	3.73 (1.45)	0–5
11 [22]	4.67 (0.99)	0–5	11 [35]	4.20 (1.35)	0–5	11 [29]	3.75 (1.38)	0–5
12 [23]	3.76 (1.59)	0–5	12 [8]	4.80 (0.69)	0–5	12 [30]	3.51 (1.25)	0–5
13 [24]	4.14 (1.37)	0–5	13 [13]	4.60 (0.81)	2–5	13 [33]	4.15 (1.36)	0–5
14 [25]	3.98 (1.37)	0–5	14 [9]	4.76 (0.68)	1–5	Total	53.74 (10.68)	1–65
Total	55.28 (11.61)	1–65	15 [23]	3.97 (1.44)	0–5			
			Total	65.27 (10.26)	26–75			

PREM-ECM-prior scores were significantly correlated with PATSAT-C33 (*r* = 0.42), HADS total (*r* =-0.31), HADS-anxiety (*r* =-0.26) and HADS-depression (*r* =-0.28). Total PREM-ECM-prior-14 total score had a weak correlation with PEQ total scale (*r* = 0.13) and a moderate correlation with PEQ subscale communication (*r* = 0.41). Test-retest reliability was assessed for the total score in 41 (25%) participants, demonstrating acceptable repeatability (*r* = 0.71). The PREM-ECM-prior demonstrated excellent internal consistency (*α* = 0.92).

Exploratory Factor Analysis presented three subscales with acceptable eigenvalues, including ‘decision making’ (*α* = 0.91); ‘support’ (*α* = 85); and ‘information needs’ (*α* =.59) (see Table 5 for eigen values).

**Table 5:** Eigen values from the Principle Component Analyses

Questionnaire	Factor	Initial Eigen-values	Initial % Variance	Eigen-values after varimax	% Variance after varimax
Prior-14	1	7.36	39.58%	3.46	23.07
	2	1.64	10.91%	2.93	19.50
	3	1.46	7.63%	2.33	15.55
On-trial-15	1	5.94	39.58%	3.46	23.07%
	2	1.64	10.91%	2.93	42.57%
	3	1.15	7.63%	2.33	56.12%
Carer-13	1	5.71%	43.94%	2.96	22.77%
	2	1.57%	12.06%	2.74	43.80%
	3	1.07%	8.13%	2.66	64.22%

##### PREM-ECM-on-trial

One hundred and sixty-two participants completed the PREM-ECM-on-trial consisting of 35 items; 14 items were removed during hierarchical reduction: ceiling effect (*n* = 8), age bias (*n* = 2), gender bias (*n* = 2), item-to-item correlation (*n* = 6), and one due to expert opinion, some items were removed for multiple reasons (see Table 6). Rasch analysis was conducted on the remaining twenty-one items. Six items were removed due to poor fit to the Rasch unidimensional model (see Table 6). The final 15-item solution (PREM-ECM-on-trial), after Bonfferoni correction, demonstrated good fit to the Rasch model (chi-square *p* = 0.02; PSI = 0.71) and good distribution of item scores (logit range: +0.0 to + 0.3).

**Table 6:** Reason for item removal for PREM-ECM-on-trial

Question Number	Question (High Score Answer)	Ceiling > 79%	gender bias	Age Correlation	Correlation with other items	Expert opinion	Rasch
6	I am given enough time to ask questions	X			X		
7	I feel comfortable asking the research team any questions I have	X					
16	The research team treats me as an individual	X			X		
3	I am able to ask the research team questions	X			X		
11	I was told what to do if I experienced side-effects	X		X			
12	I am aware I may experience side effects from the trial	X					
17	The research team gives me enough time to talk about what is important to me/my needs and priorities	X			X		
18	I feel the research team is approachable	X					
19	I am able to bring family/friends to my appointments as I want				X		
25	My family/friends receive the support they need						
26	I do not feel anxious about being on the trial		X	X			
29	The trial is well organised				X		
30	I am kept informed of delays during trial treatment		X				
32	I am still working/studying/taking part in usual activities whilst on the trial					X	
1	I have enough information about my options if the trial does not work/benefit me						X
2	I was informed that the trial might not work						X
15	I feel the research team listen to me						X
20	The research team supports me to cope with my anxieties about being on the trial						X
24	I am able to access the support available to me if I need to						X
31	I do not feel there was unnecessary waiting between consultations, tests, results, and/or pharmacy.						X

Exploratory Factor Analysis presented three subscales with acceptable eigenvalues, including ‘decision making’ (*α* = 0.83); ‘support’ (α = 0.75); and ‘Impact of trial and management of side effects (α =.70) (see Table 5 for eigenvalues).

Each PREM-ECM-on-trial is scored 0 (bad experience) to 5 (good experience). The total scores range from 0 to 75. The mean and range of scores for each of the final 15 items are presented in Table 4, with total PREM-ECM-on-trial scores ranging from 26 to 75.

PREM-ECM-on-trial significantly correlated with PATSAT-C33 (*r* = 0.47), and HADs total (*r* =-0.41) and HAD-depression subscale (*r* =-0.41) and HADs subscales-anxiety (*r* =-0.28). There was a very weak correlation with PEQ (*r* = 0.14). Test-retest reliability was assessed in 57 (35.1%) participants, demonstrating acceptable reliability (*r* = 0.76).

#### Carers

One-hundred-and-two participants completed the 34 item PREM-ECM-Carer. Five items were removed in hierarchical reduction: ceiling effect (*n* = 3), age bias (*n* = 1), item-to-item correlation (*n* = 4), (Table 7). Rasch analysis was conducted on the remaining 29 items. Sixteen items were removed due to poor fit to the Rasch unidimensional model. The final 13-item solution (PREM-ECM-Carer) demonstrated good fit to Rasch (*p* = 0.62; PSI = 0.76) and good distribution of item score.

**Table 7:** Reason for item removal for PREM-ECM-Carer

Question Number	Question (High Score Answer)	Ceiling > 79%	Age Correlation	Correlation with other items	Rasch
1	I was able to provide support with my friend/family member’s decision to participate or not in the trial	X		X	
2	My preference was that my friend/family member takes part in the trial	X			
17	I feel the research team gave us all the information they could about the side effects	X		X	
25	I feel the research team is very supportive			X	
32	Due to the trial I have not needed to reduce/change my work/ study/ leisure activities		X	X	
3	I was able to express my viewpoint on my friend/family member’s decision to participate or not				X
4	I am involved as much as I want to be with the decision to take part in the trial or not				X
5	My friend/family member and I had enough time to think about taking part in the trial				X
6	My friend/family member and I did not feel under any pressure to take part in the trial				X
7	My friend/family member and I were informed about what would be involved in trial participation				X
8	I felt that there were alternative options				X
10	The research team explained why my friend/family member could go on the trial				X
11	It was not made clear that the clinical trial may not benefit my friend/family member				X
15	I find the uncertainty around clinical trials difficult to cope with				X
20	There is not enough support…. experiences side effect/reactions				X
21	The care my friend/family member on the trial has received is personalised				X
22	Patient and I were not offered any support				X
24	feel my friend/family member on the trial has not had much support				X
27	I do not know who to contact if I need psychological/physical/financial support				X
28	My friend/family member’s participation in the trial has been a burden for me				X
31	The trial stops me from doing all my usual activities				X
34	I would not recommend others to go on a clinical trial				X

Exploratory Factor Analysis presented three subscales with acceptable eigenvalues, including ‘trial experience’ (α = 0.80); ‘burden’ (α = 0.76); and ‘support’ (α = 0.80) (see Table 5 for eigenvalues).

Each PREM-ECM-Carer is scored 0 (bad experience) to 5 (good experience). The total scores range from 0 to 65. The mean and range of scores for each of the final 13 items are presented in Table 4.

PREM-ECM-Carer-13 total score was significantly and strongly correlated with AC-QoL total score (*r* = 0.51) and AC-QoL subscale Support for caring (*r* = 0.56). It was moderately-strongly correlated with the remaining seven subscales (Caring choice subscale *r* = 0.30, Caring stress subscale *r* = 0.37, Money matters subscale *r* = 0.35, Personal growth subscale *r* = 0.30), Sense of value subscale *r* = 0.36, Ability to care subscale *r* = 0.37, and Carer satisfaction subscale *r* = 0.37).

PREM-ECM-Carer-13 total score was moderately correlated with HAD total (*r* =-0.29) and HAD-depression (*r* =-0.32), and weekly correlated with HAD-anxiety (*r* =-0.23). Test-retest reliability was assessed in 40 (39%) caregivers and was good (ICC = 0.83).

### Stage IV: pilot testing

Eleven patients completed the PREM-ECM-prior-14 (supplementary file 1), after their appointments at home. The total score ranged from 46 to 70 and the mean total score was 62.09 (standard deviation 7.05). Twenty-three patients completed the PREM-ECM-on-trial-15 (supplementary file 2), after their appointments at home. The total score ranged from 31 to 65 and the mean total score was 66.96 (standard deviation 7.43). Nineteen Careers completed the PREM-ECM-Carer-13 (supplementary file 3), at home. The total score ranged from 31 to 65 and the mean total score was 51.94 (standard deviation 11.09). Pilot testing for all three PREMs generally showed the use of PREMs were feasible in capturing both patient and caregiver experiences within routine clinical environments.

## Discussion

This study is the first to report the development and validation of a PREM for patients and carers of patients participating in early-phase cancer clinical trials. The importance of collecting patient experience data in clinical practice has been highlighted in a number of research studies. A review of patient experience and the association to clinical outcomes reported that there is evidence of correlations between patient experience, clinical effectiveness and patient safety across a range of different disease groups [35]. Evidence suggests that improving patient experience can have a positive impact on many clinical outcomes, therefore it is important to ensure that patient experience is measured in some way [36, 37]. A UK Department of Health report has suggested that improving patient experience can lead to improvements in both effectiveness and safety [38].

The aim of this study was to develop a single PREM however early qualitative work with patients identified the need for two questionnaires that captured experiences at different points in the clinical trial pathway and one that captured carers experience throughout the trial. The first questionnaire (PREM-ECM-Prior) captures the initial information the patient received about the trial and their understanding of the information, decision making regarding trial participation, expectations, and support during the trial screening phase and early trial participation (< 6 weeks). The second questionnaire (PREM-ECM-on-trial) captures the experience of being a trial participant, decision making regarding continuing/withdrawing from the trial, side effects and support. The pilot study indicated it would be feasible to collect this data in routine clinical practice. The third questionnaire (PREM-ECM-Carer) captures the experience of family and friends of those on a clinical trial.

Each of the PREM-ECMs demonstrated a good-fit according to the RASCH model which suggests that the items relate to the same underlying structure (patient and carer experience) and allows an overall experience score to be obtained from the total score. Each of the PREM-ECMs demonstrated an underlying pattern of three sub-domains. The pattern of sub-domains mirrored our presentation of the qualitative results and reflects the core themes identified.

Both PREM-ECMs were moderately correlated with the PATSAT-C33 [28] questionnaire, which was developed to capture cancer patients’ experiences, suggesting the PREM-ECM questions capture patients’ experiences but different patient perspectives from the PATSAT-C33 measure. The PREM-ECM on trial and PREM-ECM-Carer was also moderately correlated with the HADS [29]. Other research in primary care has shown associations between patient satisfaction and anxiety and depression, the study found that patients who were least satisfied with their healthcare provision were more likely to experience anxiety and depression [39]. While on the trial, patients will likely experience side effects and receive treatment prognosis/updates, which could explain why the on trial questionnaire correlated more strongly compared to the prior questionnaire.

The PREM-ECM questionnaires and the PEQ [19] were weakly correlated, suggesting the PREM-ECM is capturing a different patient experience. This finding is not unexpected given that the PEQ was developed for primary care and the PREM-ECM questionnaires were developed for tertiary care and specific to clinical trial experience. Contact with General Practitioners in primary care is a patient’s first contact for health concerns whereas patients seeking care at a cancer centre are at very different stage of the illness trajectory and therefore would have very different experiences. Some questions from the PEQ may not have been appropriate for patients on early phase clinical trials. For example the question “Do you know what to do to reduce or prevent your health problem” may not be relevant as patients on clinical trials have often exhausted all others lines of treatment and there it is likely they can do very little to ‘prevent or reduce’ their cancer. Similarly, the question “Will it lead to fewer health problem(s)? Or help prevent problems?”, may not be appropriate as when patients commence a trial they are presented with a long list of potential side-effects therefore it is somewhat uncertain as to whether the treatment will lead to fewer health problems. In some cases, it may be the opposite and patients may experience more problems as a result of participating in the trial. The majority of patients taking part in clinical trials are aware the trial may not benefit them so there was no guarantee it would “reduce their health problems” [6]. When the PREM-ECM questions were compared with the communication subscale of PEQ, both questionnaires were moderately correlated with the subscale, suggesting some of the other subscales may not have been relevant for ECM patients and highlight the importance of having a PREM specific to clinical trials.

The PREM-ECM-Carer questionnaire was strongly correlated with the AC-QoL, a validated questionnaire, which measures adult carers’ quality of life and the subscale caring for adults. This strong correlation suggests it is capturing carers’ experiences, the moderate to weak relationship between the other subscale suggests our questionnaire is capturing a slightly different carer experience. This finding is consistent with our expectation, as our questionnaire is tailored specifically for individuals caring for ECM patients. In contrast, the AC-QoL is designed for caregivers in a broader context, encompassing personal benefit and the sense of feeling valued. Interestingly, these particular facets were not mentioned in our interview data, reinforcing the relevance and necessity of our specialised questionnaire for ECM caregivers. Particularly as family and friends of cancer patients may find themselves acting in a caregiver role, for example they may manage appointment schedules, manage medication and side effects from treatment, and provide practical care and/or emotional support. In addition their families may be required to put some parts of their lives on hold or make alterations to their daily routine, hobbies, and/ or social life [9, 10]. Caregivers can also experience distress and anxiety [8, 11, 40, 41]. Untreated anxiety and depression can result in poor physical and mental health, which may reduce both the caregiver and their family member with cancer quality of life and care their family receive [42–44]. By using the PREM-ECM-Carer-13, it can identify areas carers need extra support, which may lead to better quality of life and improve the care family members receive.

Both the patient and carer questionnaires demonstrated excellent-good internal consistency suggesting the questionnaire measures what it is designed to capture. Both questionnaires demonstrated acceptable test-retest reliability, suggesting the questionnaire had good test consistency across the two time points. The PREMs can be used in clinical practice as an indicator of the quality of care from the patient’s perspective and used to audit and improve the care received and compare care across hospitals. In order to improve care the successful implementation of PREMs in service is crucial. However, achieving this implementation is not without challenges [45]. Key obstacles include addressing the time constraints faced by healthcare professionals, who must efficiently collect and record the data derived from PREMs. Furthermore, overcoming the increased workload associated with these tasks is essential for seamless integration into daily practice. The use of digital PREMs may reduce some of the additional workload in collating data. Furthermore, resistance and reluctance among some healthcare staff and the perception of an added burden on patients pose additional hurdles [31]. During the Focus groups patients’ unanimously agreed the need for two PREMs and the continued use of PREMs throughout the trial process. HCPs, when made aware of this patient-driven advocacy, may be more inclined to view PREMs not as an additional burden but as a valuable tool for enhancing patient experiences and overall quality of care. Additionally emphasising achievements, not just the improvements, on a regular basis, may increase utilisation of PREMs among HCPs [45].

Additionally the PREMs can be used to aid decision making. One of the main themes from the interviews and focus groups, was around the decision-making process regarding participating in a clinical trial and their treatment choice or perceived lack of it. For many, clinical trials represented their final source of hope. With some misunderstandings about dose level and side effects. This is consistent with previous studies, which found trial patients believed higher doses were more effective and side effects were caused by effective treatment [6, 46, 47]. Furthermore, the inadequacy of information emerged as a recurrent barrier to effective decision-making, echoing previous studies [6, 33]. Previous research revealed that healthcare professionals (HCPs) play a crucial role in shaping decisions and influencing the decision-making process, and HCPs preformed decisions often lead to patients perceiving a diminished sense of choice [33]. However, when HCPs actively inquired about patients’ expectations, concerns, and hopes, it fostered discussions towards treatment options aligned with patient preferences [33]. Gregersen et al. [48] highlight the importance of HCPs comprehending patient concerns and preferences. The PREMs provide an opportunity to facilitate shared, informed decision-making between patients and HCPs. As such, the integration of PREMs in clinical settings holds promise for enhancing the collaborative decision-making process, ensuring a more patient-centered approach throughout the clinical trial.

### Limitation

All cognitive interviews were conducted with white males, which is not representative of the UK clinical trial population. The study also only recruited patients who could comprehend fluent English; therefore the questionnaires may not be applicable across a diverse patient group. This study was performed in a single cancer centre and therefore may not be representative of the experiences of patients from other cancer centres, however, patients were recruited across all disease groups to improve the representativeness of patients’ experiences. The study did not identify the optimal time and frequency for the questionnaires to be administered, this would need to be identified in future studies.

## Conclusion

This study has outlined the development and preliminary validation of three patient reported experience measures, to capture patients’ and their carers’ experience of ECM trials, which could be used alongside routine clinical trial, to provide indicators for change and capture quality improvements. Further studies are required in larger, multicentre cohorts to further test the validity of the questionnaire.

### Electronic supplementary material

Below is the link to the electronic supplementary material.

Supplementary Material 1

Supplementary Material 2

Supplementary Material 3

Supplementary Material 4

Supplementary Material 5

Supplementary Material 6

Supplementary Material 7

Supplementary Material 8

Supplementary Material 9

Supplementary Material 10

## Data Availability

The data that support the findings of this study are available on request from the corresponding author. The data are not publicly available due to privacy or ethical restrictions. The study is registered on clinicaltrials.gov https://clinicaltrials.gov/ct2/show/NCT05386602 (NCT identifier: NCT05386602).

## Abbreviations

AC-QoL: Adult Carer Quality of Life Questionnaire
COPD: Chronic Obstructive Pulmonary Disease
ECM: Experimental Cancer Medicine
EORTC: European Organisation for Research and Treatment of Cancer
GRAMMS: Good Reporting of A Mixed Methods Study
HADS: Hospital Anxiety and Depression Scale
ICCC: Intra-Class Correlation Coefficient
PATSAT-C33: Patient satisfaction with cancer care questionnaire
PCA: Principals Components Analysis
PEQ: Patient Experience Questionnaire
PREM: Patient Reported Experience Measure
SPSS: Statistical Package for Social Sciences
