# Supplementary material for: Development and validation of a patient reported experience measure for experimental cancer medicines (PREM-ECM) and their carers (PREM-ECM-Carer)
Source: BMC Cancer. 2024 Apr 19;24:500. doi: 10.1186/s12885-024-11963-x (PMC11031988; doi:10.1186/s12885-024-11963-x)
Supplement: Supplementary file 1 — Supplementary Material 1 [file 12885_2024_11963_MOESM1_ESM.doc]

Supplementary_file_1_PREM-ECM prior_14items_final

This questionnaire will help us to understand the experience of our patients who have been given information about taking part in an experimental cancer trial. On each side of the numbers there is a statement. Please answer every question by placing a tick over the **ONE NUMBER** that best describes your experience of your experimental cancer trial.

Example: If you felt you had very little energy you would tick 1 (see below)

I have no energy at all 1 2 3 4 5 I have lots of energy


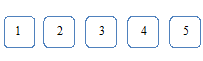


1 [1]. I feel the information about the trial is not presented at the right level for me to understand

I feel the information about the trial is presented at the right level for me to understand


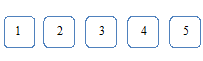


2 [11]*. I feel my questions are not fully answered

I feel my questions are fully answered


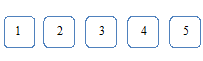


3. [4] I was not informed that the trial might not work

I was informed that the trial might not work


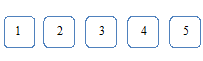


4. [5] The possible side effects have not been clearly explained to me

The possible side effects have been clearly explained to me


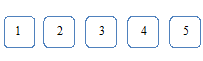


5. [3] I would like more information about my other options if I choose not to take part in the trial

I have enough information about my other options if I choose not to take part in the trial


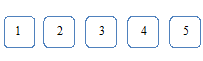


6*. [19] My family/friends are not as involved as I want them to be with the decision to take part in the trial

My family/friends are as involved as I want them to be with the decision to take part in the trial

7. [12] I am not aware that my case has been discussed with other health care professionals

I am aware that my case has been discussed with other health care professionals


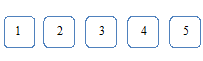


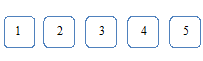


8. [13] I have not been given the opportunity to discuss the clinical trial with other members of the research team

I have been given the opportunity to discuss the clinical trial with other members of the research team


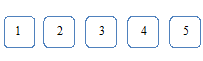


9*[22]. I do not know whom to contact and how if I need to

I know whom to contact and how if I need to


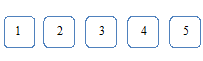


10 [20]. I feel the research team does not listen to me

I feel the research team listens to me


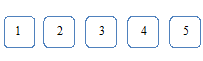


11*[24]. If I need emotional support I do not know how to access it

If I need emotional support I know how to access it

12.[25] The research team did not support me to cope with my anxieties while waiting to see if I was eligible

The research team supported me to cope with my anxieties while waiting to see if I was eligible


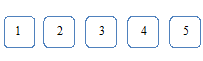


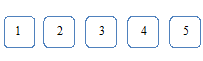


13*.[10] My family/friends are unable to ask the research team questions

My family/friends are able to ask the research team questions


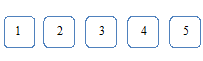


14. [23] My family does not know how to access emotional support

My family knows how to access emotional support
