# Supplementary material for: Development and validation of a patient reported experience measure for experimental cancer medicines (PREM-ECM) and their carers (PREM-ECM-Carer)
Source: BMC Cancer. 2024 Apr 19;24:500. doi: 10.1186/s12885-024-11963-x (PMC11031988; doi:10.1186/s12885-024-11963-x)
Supplement: Supplementary file 2 — Supplementary Material 2 [file 12885_2024_11963_MOESM2_ESM.doc]

Supplementary file 2. PREM-ECM On trial 15 Items final

This questionnaire will help us to understand the experience of our patients who have been given information about taking part in an experimental cancer trial. On each side of the numbers there is a statement. Please answer every question by placing a tick over the **ONE NUMBER** that best describes your experience of your experimental cancer trial.

Example: If you felt you had very little energy you would tick 1 (see below)

I have no energy at all 1 2 3 4 5 I have lots of energy


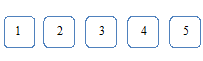


1*[5]. I feel my questions are not fully answered

1. I feel my questions are fully answered

2* [4]. My family/friends are unable to ask the research team questions


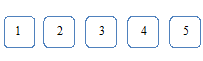


My family/friends are able to ask the research team questions

3.[10] I feel uncomfortable letting the research team know about side effects

I feel comfortable letting the research team know about side effects


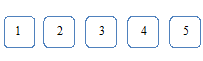


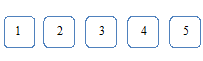


4. [14] I do not know how to manage/treat side effects

I know how to manage/treat side effects


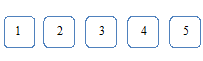


5*. [21] I do not know whom to contact and how if I need to

I know whom to contact and how if I need to


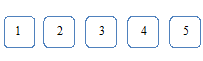


6*. [22] If I need emotional support I do not know how to access it

If I need emotional support I know how to access it


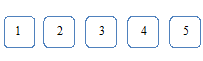


7. [27] The research team was not able to be flexible around the delivery of my treatment

The research team was able to be flexible around the delivery of my treatment


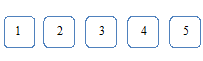


8. [28] Any issues with the clinical trial were not resolved

Any issues with the clinical trial were resolved

33. I feel the trial controls my life

I feel the trial does not control my life


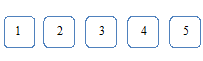


9. [33] I feel the trial controls my life

I feel the trial does not control my life


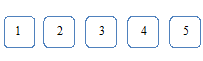


10. [34] It is difficult to fit working/studying/ usual daily activities around the trial

It is easy to fit working/studying/ usual daily activities around the trial


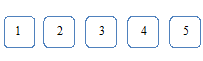


11. [35]Participating in the trial does affect me financially

Participating in the trial does not affect me financially


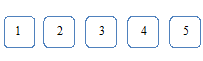


12. [8] I do not feel able to come-off / withdraw from the trial if I want to

I feel able to come-off / withdraw from the trial if I want to


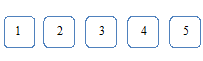


13. [13]I am not willing to continue in the trial if I experience side effects

I am willing to continue in the trial if I experience side effects


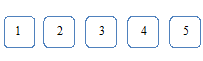


14*. [9] My family/friends are not as involved as I want them to be with the decision to continue to stay on the trial

My family/friends are as involved as I want them to be with the decision to continue to stay on the trial


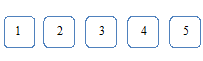


15. [23] My family does not know how to access emotional support

My family knows how to access emotional support
