# Supplementary material for: Development and validation of a patient reported experience measure for experimental cancer medicines (PREM-ECM) and their carers (PREM-ECM-Carer)
Source: BMC Cancer. 2024 Apr 19;24:500. doi: 10.1186/s12885-024-11963-x (PMC11031988; doi:10.1186/s12885-024-11963-x)
Supplement: Supplementary file 3 — Supplementary Material 3 [file 12885_2024_11963_MOESM3_ESM.doc]

This questionnaire will help us to understand the experience of the family and friends of our patients who are taking part in an experimental cancer trial. On each side of the numbers there is a statement. Please answer every question by placing a tick over the **ONE NUMBER** that best describes your experience of having a family member or friend on an experimental cancer trial.

Example: If you do not enjoy reading books at all tick 0 (see example below)

I enjoy reading books

I do not enjoy reading books

0 1 2 3 4 5


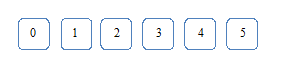


1. [9] I was not fully prepared/aware of what participating in the trial would involve (e.g. number of visits)

I was fully prepared/aware of what participating in the trial would involve (e.g. number of visits)


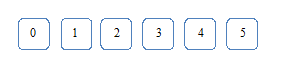


Any issues my friend/family member and I experienced on the trial were resolved

2. [12] Any issues my friend/family member and I experienced on the trial were not resolved


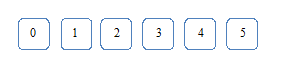


3. [13] My friend/family member is not always truthful about their symptoms/side effects during consultations

My friend/family member is always truthful about their symptoms/side during consultations


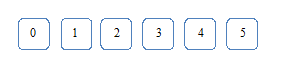


4. [14] I find it difficult to disclose information about my friend/family member’s symptoms/side effects to research team

I find it easy to disclose information about my friend/family member’s symptoms/side effects to research team


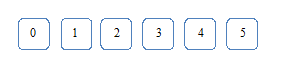


5. [16] My friend/family member and I are not kept informed/updated regularly of their trial progress/test results

My friend/family member and I are kept informed /updated regularly of their trial progress/test results


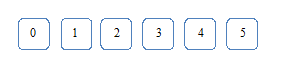


6. [18] I was not aware that the side effects of the trial could be severe

I was aware that the side effects of the trial could be severe


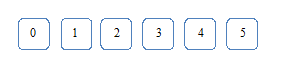


7. [19] I feel I cannot manage my friend/family member’s side effects

I feel I can manage my friend/family member’s side effects


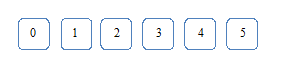


8. [22] My friend/family member and I were not offered any support (e.g. practical and/or emotional)

My friend/family member and I were offered support (e.g. practical and/or emotional)


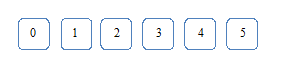


10. [26] I feel I do not have enough support (e.g. Maggie’s/Macmillan nurses/GP/ Community services/other supports)

9. [23] If I needed support I would not know how and who to ask for support

If I needed support I would know how and who to ask for support


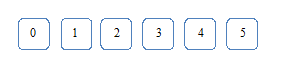


I feel I have enough support (e.g. Maggie’s/Macmillan nurses/ GP/ Community services/other supports)


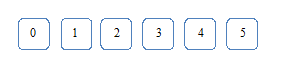


11. [29] I feel the trial has taken over my life

I do not feel the trial has taken over my life at all


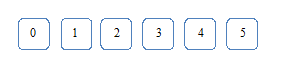


12. [30] Due to the trial I feel that I have put my personal life on hold

Due to the trial I feel that I have not put my personal life on hold at all


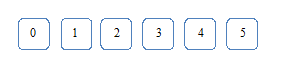


13. [33] Trial participation has impacted me financially

Trial participation has not impacted me financially at all
