# Supplementary material for: Development and validation of a patient reported experience measure for experimental cancer medicines (PREM-ECM) and their carers (PREM-ECM-Carer)
Source: BMC Cancer. 2024 Apr 19;24:500. doi: 10.1186/s12885-024-11963-x (PMC11031988; doi:10.1186/s12885-024-11963-x)
Supplement: Supplementary file 4 — Supplementary Material 4 [file 12885_2024_11963_MOESM4_ESM.docx]

*Supplementary Table 1.* ***Caregiver demographic characteristics of participants for each stage of the study***

|  | | **Stage I** | **Stage II** | **Stage III** | **Stage iv** |  |
| --- | --- | --- | --- | --- | --- | --- |
|  |  | Interviews (*n*=10) | *Cognitive interview (n*=3) | PREM-ECM Carer *(n*=102) | PREM-ECM Carer-13 (*n*=19) |  |
| **Age Mean (SD)** | | 51.33 (19.76) | 72.50 (2.12) | 61.23 (12.14) | 60.00 (15.25) |  |
| **Gender (Male)** %(*n* | | 16.67% (1) | 66.67% (2) | 33.70% (34) | 36.8% (7) |  |
|  | | | | |  |  |
| **Ethnicity** | White British | 83.33% (5) | 100% (3) | - | - |  |
|  | Chinese | 16.67% (1) | - | - | - |  |
|  | | | | |  |  |
| **Marital status** | Single | 16.7% (1) | - | - | - |  |
|  | Married/domestic partner | 83.3% (5) | 100% (3) | - | 73.68% (14) |  |
|  |  | | | |  |  |
| **Employment status** | Employed | 20% (1) | 33.3% (1) | 38.0% (38) | 33.3% (6) |  |
|  | Student | 20%% (1) | - | - | - |  |
|  | Retired | 40% (2) | 66.7% (2) | 60% (60) | 55.56% (10) |  |
|  | Volunteer | 20% (1) | - | - | - |  |
|  | Unemployed/unable to work | - | - | 2.0% (2) | 11.11% (2) |  |
|  | | | | |  |  |
| Relationship to patient | Wife/partner | 40% (2) | 66.67% (2) | 44.00% (44) | 38.89% (7) |  |
|  | Husband/partner | - | 33.33% (1) | 36.00% (36) | 38.89% (7) |  |
|  | Sibling | 20% (1) | - | 7.00% (7) | 5.56% (1) |  |
|  | Child | 40% (2) | - | 5.00% (5) | 11.11% (2) |  |
|  | Parent | - | - | 3.00% (3) | 5.56% (1) |  |
|  | Friend | - | - | 3.00% (3) | - |  |
|  | Niece | - | - | 2.00% (2) | - |  |
|  | | | | | | |
| Phase of trial | Phase 1 | 50% (3) | - | 61.76% (63) | 68,4% (13) | |
|  | Phase 2 | 50% (3) | - | 38.24% (39) | 31.60% (6) | |
