# Supplementary material for: Development and validation of a patient reported experience measure for experimental cancer medicines (PREM-ECM) and their carers (PREM-ECM-Carer)
Source: BMC Cancer. 2024 Apr 19;24:500. doi: 10.1186/s12885-024-11963-x (PMC11031988; doi:10.1186/s12885-024-11963-x)
Supplement: Supplementary file 5 — Supplementary Material 5 [file 12885_2024_11963_MOESM5_ESM.docx]

Supplementary Table 2. The themes and their sub-themes from patient interviews and focus groups with supporting quotations

| **1. Decision making** | |
| --- | --- |
| 1.1 Decision maker | *“It was mine [decision]. It’s got to be, it’s my life.”(25M83)*  *“I think the consultants made the decision about what was most suitable.”(28F72)*  *“The whole family read it, all my children, my husband. We discussed it, come back and said yes.” (07F61)* |
| 1.2 No other option | *“I had no other choice, so at the end of the day, that’s the one.”(25M83)* |
| 1.3 Hope of a cure | *“There’s that hope there that the…a chance of a cure.”(19M56)* |
| **2. Information needs** | |
| 2.1 Volume & simplicity of information | *“doctors know all the technical terms, but we don’t, most of us; and it has to be said in plain English”(22F52)* |
| 2.2 Side effects | “I don’t remember anybody saying, before you go on this, your thyroid could do this, and it will be permanent”(23F68) “Well I think the list of possible side effects is they just think of everything they can think of that might go wrong and list them all down” (FG2) |
| 2.3 Updates throughout treatment | *“I want him to say to me, now look here, if this doesn’t work it's chemo.”(21F70)* |
| 2.4 Provision of False hope | “if they hadn’t built my expectation, then the crash wouldn’t have been as hard” (03M42) |
| 2.5 Support available | “you do feel that there's a void like, you know, where do I get that information [about support].”*(FG2)* |
| **3. Experience of trial** | |
| 3.1 Patient centred | “the clinical trials team were I felt tailoring their treatment of me.”(24F56)  “it didn’t feel personal; it felt as though I was being treated as a number that was insignificant.”(22F52) |
| 3.2 Disclosing side effects | “is the most frightening thing, at that point in time when you come off that point when you've been given, this is your one hope to live and somebody says, I'm just going to take it away from you, and that's the end of the matter.” (FG2)  “and I made the mistake of telling them about some of the side effects” (FG2)  “if you don't tell them [side effects], then you're compromising not only the trial but you're compromising yourself more importantly.”(FG2) “if they [trial team] said to you that if you were to disclose the side-effects you're having, that they would be more likely to change your treatment levels or do something about it, other than say on or off because it's the fear of the on or off is the most frightening thing”.(FG2) |
| **4. Impact of trial participation** |  |
| 4.1 Quality of Life (QoL) | “It’s an impact on your life having to come in every two weeks, especially the thing I was on initially was an all-day effort” (FG2)  “I was in and out like a yo yo And I didn’t realise it was the trial”(14M66) “Our life has changed absolutely beyond recognition. I had a good job and we were very active, cycled everywhere and went diving on holiday and all of those things which we can’t do now“(24F56) |
| 4.2 Time | “the frequency of the visits is good and bad, as I say It’s travelling every week but having that line of contact and support weekly is great.” (24F56) |
| 4.3. Family | “It’s a really valid question to ask around carers and family members and how are they coping”(26F48)  “it’s difficult for my daughter because she had her studies and she came with me every time”(29M55) |
| 4.4 Financial | “It costs you a lot of money”(07F61) |
| 4.5 Psychological impact | “Depressed. it got me down, the waiting”(28F72) “So I’m on the waiting list. They may pick somebody else. I don’t know.” (25M83) |
