# Supplementary material for: Development and validation of a patient reported experience measure for experimental cancer medicines (PREM-ECM) and their carers (PREM-ECM-Carer)
Source: BMC Cancer. 2024 Apr 19;24:500. doi: 10.1186/s12885-024-11963-x (PMC11031988; doi:10.1186/s12885-024-11963-x)
Supplement: Supplementary file 6 — Supplementary Material 6 [file 12885_2024_11963_MOESM6_ESM.docx]

Supplementary Table 3 The themes and their sub-themes from carer interviews with supporting quotations

| **1. Decision making** | |
| --- | --- |
| 1.1 Decision maker | *“as involved as we can be really, yes, there was… I mean, we ultimately gave Mum the final say so and we all said that, to respect her needs. She is the patient”. (CA015)*  *“I just wanted to support him in whatever decision he made, that was all it was really.” (CA102)*  *“And they didn’t really want to say [how long he had left]. You know. And he eventually said, because he was persistent, and he eventually said, we would expect within a few months that you would be experiencing problems. , I have to admit at that point, I was sort of thinking, why go through more treatment? Why not, if you have only got a few months” (CA042)* |
| 1.2 No other option | *“Thankful, it was the only option…it’s an option that not everybody gets or not everybody knows about, so we were grateful because we didn’t have an option the week before, so it was a blessing.” (CA031)*  *“It was an option open to us and it was one that we wanted to explore. We’d been down the chemotherapy route, as I said, and that didn’t do anything for him, so the clinical trial just seemed the right way forward for us at the time.” (CA041)* |
| 1.3 regret | *“I don’t know whether it would have swayed us one way or the other, knowing…you know, put us off if we’d have known what it involved, or whether we’d have still been happy to do it,” (CA102)*  *“a couple of times from 2016, I’ve kind of felt that would we have been better not doing it? Would we have more time together?” (CA101)* |
| 1.4 time to decide | *“when the cancer came back he had had time to think about it, so he decided that he would like to do it.” (CA10)* |
|  |  |
| **2. Information needs** | |
| 2.1 Volume & simplicity of information | *“it was very straightforward really. It explained exactly what would happen, to expect to be there on a treatment day, all day really, when you would have scans, whether on not he could, whether there might be a reason why he couldn’t go on the trial, because, it wasn’t certain”* (CA042) |
| 2.2 Side effects | “I don’t think they could have given us any more information than they did; I think it’s an unknown quantity on how the patient reacts, so how it affected Name. But no, I think we were given the information that was necessary. *(CA102)* |
| 2.3 Updates throughout treatment | “They’ve been brilliant in that they’ve kept us informed on everything” (CA102) |
| 2.4 Support available | “Yeah. I mean, they are incredibly supportive here. …..So I know the support is there if I needed it” (CA041)  “Yeah,you’ve got all the information. You get all the information don’t you, and you’ve got all the contact numbers that you need should you need them, and if you haven’t got anything you just ask and then somebody will sort it for you.” (CA042) |
| **3. Experience of trial** | |
| 3.1 managing side effects | “Yes, the side-effects affected myself and my dad the most because we never really knew how severe they were going to be.” (CA015)  “There’s obviously the hotline but then there’s no transportation and there’s no beds at the ward so like nothing matched up. Then we’d spend seven hours at an A&E that needed for the clinical team department to wake up so that they could call them about the drugs that she can take. (CA012) |
| 3.2 Disclosing side effects | “he didn’t tell the truth either, you see, he’d say things, he was fine and all the rest of it, but then all week he’d been off or he’d been complaining to me about things; and I think slowly but surely I’m saying, well, no, that’s not quite true, you did have a…you know, but unless I make a different appointment and come, and talk to somebody, I find that difficult.” (CA10) |
| **4. Impact of trial participation** |  |
| 4.1 Quality of Life (QoL) | “but, yes, it is a burden and no matter what my parents keep saying to me, it’s like, oh, you don’t need to worry about us. It’s like, I live with you. There’s no chance of me ignoring what’s happening downstairs when someone’s coughing their guts out or whatever. It’s not like you can pretend it’s not happening so, yes, it’s been a bit of a burden * (CA015)  “’I’ve stopped doing quite a bit because of the brain tumour, because I was worried about him having seizures and things like that.” *(CA102)* |
| 4.2 Work | “I took partial retirement, so I only work two days a week” *(CA042)* |
| 4.3. Relationship with Family | “he brings nearly every conversation round something to do with his illness, instead of controlling the cancer he’s letting cancer control him” *(CA102)*  *“And I don’t have time to go away with my wife anymore because I haven’t go the holidays, unless I take them off unpaid,” (CA101)* |
| 4.4 Financial | “because I have struggled financially, various things because I’ve had to turn down work so that I can stay at home for Mum.”  *(CA015)*  “I didn’t know, only very recently, that I had the option of applying for carer allowance or finding free or subsidised care stuff to help my parents go out for a meal and stuff like that.” *(CA015)* |
| 4.5 Psychological impact | “because I was virtually caring for him 24/7 when he was really ill and it was just awful to watch somebody go down to a 12 stone bag of bones. He was 16 stone.” *(CA041)* |
| 4.6 Time | “if I’m totally honest, we weren’t prepared for the amount of visits we had to make to hospital.” (CA102) |
