# Supplementary material for: Development and validation of a patient reported experience measure for experimental cancer medicines (PREM-ECM) and their carers (PREM-ECM-Carer)
Source: BMC Cancer. 2024 Apr 19;24:500. doi: 10.1186/s12885-024-11963-x (PMC11031988; doi:10.1186/s12885-024-11963-x)
Supplement: Supplementary file 7 — Supplementary Material 7 [file 12885_2024_11963_MOESM7_ESM.docx]

Figure 1. Study schema

**Stage I (item generation)**

*Patients: n*=22 interview *& n*=12 Focus group Carers: *n*=10 interview

**Stage II (Cognitive interview)**

*Patients: n*=5 (1 naïve, 4 who participated in stage I) reviewed the draft PREM-ECM-prior & on-trial

Carers: *n*=3 (all naïve) reviewed draft PREM-ECM-Carer

**Stage III (Item reduction and refinement Time 1)**

Patient: *n*=162 PREM-ECM-prior

*n*=162 PREM-ECM-on trial

All 324 completed

- EORTC PATSAT-C33
- PEQ
- HADS

Carers: *n*=102

- Draft PREM-ECM-Carer
- AC-QoL
- HADS

**Stage III Time 2 (Time 1 +7days)**

*Patients:*

- *n*= 54 Draft PREM-ECM (prior)
- *n= 66 Draft PREM-ECM (on trial*

*Carers:*

- *n*= 47 Draft PREM-ECM-Carer

**Stage IV (Pilot testing)**

Patients: *n*=11 Final PREM-ECM prior-14 & *n*=23 Final PREM-ECM on-trial-15

Carers: *n*= 19 Final PREM-ECM-Carer-13
