# Supplementary material for: Development and validation of a patient reported experience measure for experimental cancer medicines (PREM-ECM) and their carers (PREM-ECM-Carer)
Source: BMC Cancer. 2024 Apr 19;24:500. doi: 10.1186/s12885-024-11963-x (PMC11031988; doi:10.1186/s12885-024-11963-x)
Supplement: Supplementary file 8 — Supplementary Material 8 [file 12885_2024_11963_MOESM8_ESM.docx]

Appendix 1. Good Reporting of A Mixed Methods Study (GRAMMS)

| Guideline | Section | page |
| --- | --- | --- |
| Describe the justification for using a mixed methods approach to the research question | Methods- study design | Page 4, Lines 21-24 |
|  | | |
| Describe the design in terms of the purpose, priority and sequence of methods | Methods- study design | Figure 1  Page 4, Lines 20-24 |
|  | | |
| Describe each method in terms of sampling, data collection and analysis | Method study design  Stage I:item generation  Stage II: Cognitive debriefing  Stage III: Item reduction  Stage IV: Pilot testing  Data analysis | Page 4, lines 25-30  Page 5, lines 8-11, 16-17  Page 5 lines 28-29 31 page 6 1-8,  Page 6, lines 11-19 page 7 lines 9-12, lines 15-18.  Page 7 lines 26-29  Page 7 31-32, page 8 lines 1-31 |
|  | | |
| Describe where integration has occurred, how it has occurred and who has participated in it | Results Stage I: Item generation  Stage II: Cognitive debriefing  Stage III: Item reduction, PREM-ECM-prior  PREM-ECM-on-trial | Page 10, lines 4-12  Page 11, lines 15-19, lines 21-23  Page 12 lines 1-7  Page 13, lines 21-26  Page 14, lies 44-48 |
|  |  |  |
| Describe any limitation of one method associated with the present of the other method | Not mentioned | Not mentioned |
|  |  |  |
| Describe any insights gained from mixing or integrating methods | Discussion | Page 19, lines 208-209, page 20, lines 210-211 |
