# Supplementary material for: Development and validation of a patient reported experience measure for experimental cancer medicines (PREM-ECM) and their carers (PREM-ECM-Carer)
Source: BMC Cancer. 2024 Apr 19;24:500. doi: 10.1186/s12885-024-11963-x (PMC11031988; doi:10.1186/s12885-024-11963-x)
Supplement: Supplementary file 9 — Supplementary Material 9 [file 12885_2024_11963_MOESM9_ESM.doc]

**Questions**

**Pre Trial**

1. At diagnosis, how were you informed about your illness?

- Was there anything that could have been done differently?

1. What was your understanding of the treatment?

- Was there a discussion about different options for treatment?
- Was there a discussion about if the treatment didn’t work, how was that approached?

1. How were you involved in decisions about your treatment?
2. How did you feel after your first meeting with the doctors at the Christie?

- Was this for a trial or standard of care?
- Were you seen by different teams for trial and non-trial visits?
- If so how were the visits different?

1. How was the subject of a clinical trial approached?

- At what point was this done?
- Was the timing appropriate for you?

1. Was there a discussion about side effects and management?

- How was this approached?
- What advice and information was given?
- Did this change your decision making and how?

1. Was there a discussion about supportive care?

- When did this take place?
- What was discussed?
- How did this make you feel?
- Would you like this to have been approached differently or at a different time?
- How did this information impact your decision making going onto a trial?

1. Were your family and friends involved if you wanted them to be?
2. Was there anything you would have like to be prioritised more?
3. When you were told that a trial was the next option, how were you supported?

- How was the care pathway explained to you?
- Was it consistent across all members of staff?
- Was the pathway updated to suit your needs? Can you give an example?
- What could have been done differently?

1. What was your impression of communication between your clinical team?

- Was everyone you spoke to aware of your care path?

1. Was everything explained to you in a way that you understood, how was this done?
2. Were you given time to ask questions?

- How did you feel about asking questions?

1. Did you know who to contact if you had questions or needed support?

- How were you told about this?
- Who were you told to contact?
- What information were you given about when and why to contact?

1. Going onto a clinical trial, what were your expectations?

- What did you expect from the treatment?
- What did you expect from your doctors and nurses?
- Were these expectations met?

1. Did the care you receive feel personal?

- What was/could have been done to make it feel personal?

1. Was there anything that detracted from your care?
2. How were the visits organised?

- Did they run on time?
- What were some problems you encountered?
- Were you waiting for long periods of time?
- If so were you kept informed?
- Do you think this would have been different if you weren’t on a trial and how?

1. In a questionnaire – what questions would allow you to get your experience across?

- What would you like to be/have been asked?

1. What would you like to see change?
2. Based on your experience, would you go onto another trial in the future and why?
3. *Do you worry about your carers?*

*What support do you think they need?*
