# Supplementary material for: Development and validation of a patient reported experience measure for experimental cancer medicines (PREM-ECM) and their carers (PREM-ECM-Carer)
Source: BMC Cancer. 2024 Apr 19;24:500. doi: 10.1186/s12885-024-11963-x (PMC11031988; doi:10.1186/s12885-024-11963-x)
Supplement: Supplementary file 10 — Supplementary Material 10 [file 12885_2024_11963_MOESM10_ESM.doc]

**Questions**

1. Prior to meeting with the trials team at the Christie, what were your perceptions about clinical trials?

- How did these perceptions make you feel?
- How did these perceptions come about?
- Where had you got the information from?

2. How was the decision for your significant other/family/friend to participate in a clinical trial made?

- Were you as involved in the decision making as you wanted to be?
- Was your significant other involved as much as they wanted them to be?
- Was there anything you or your significant other would have wanted more information about when making your decision?

3. Can you tell me about the discussion about side effects and management?

- What advice and information was given?
- Was everything explained to you in a way that you understood?
- How was this done?

4. Was there a discussion about if the treatment didn’t work?

- How did that make you feel?
- Was everything explained to you in a way that you understood? How was this done?
- Were you given time to ask questions? Were you happy with the way your questions were answered?

5. Prior to being on a trial, what effect did the cancer have on your life and how is this different to being on the trial?

- What would you say has had the biggest impact?
- What effect did the trail have on your significant other

6. Trial schedules can be very intense, what effect did this have on you

- Did family and friends help with attending visits?

- How did you feel about helping your significant other/family/friend?

7. Have you had to help manage any side effects or other issues whilst on the clinical trial?

- How did side effects affect you

8. Were you able to communicate with your clinical team?

- If you had concerns were people willing to listen

- Were you kept informed at all points and how?

- Did you know who to contact if you had questions or needed support?

9. Did you feel you had support?

- would you have liked more support from family and friends?

- Support from employers, clinical care team, or psychological support

10. Based on your experience on the clinical trial, what could be done better/differently?

11. In a questionnaire – what questions would allow you to get your experience across?

- What would you like to be/have been asked?

12. Is there anything you would like to add that we’ve not already covered?
